# Proximal Tubule Dysfunction Is Associated with Podocyte Damage Biomarkers Nephrin and Vascular Endothelial Growth Factor in Type 2 Diabetes Mellitus Patients: A Cross-Sectional Study

**DOI:** 10.1371/journal.pone.0112538

**Published:** 2014-11-14

**Authors:** Ligia Petrica, Adrian Vlad, Gheorghe Gluhovschi, Florica Gadalean, Victor Dumitrascu, Cristina Gluhovschi, Silvia Velciov, Flaviu Bob, Daliborca Vlad, Roxana Popescu, Oana Milas, Sorin Ursoniu

**Affiliations:** 1 “Victor Babes” University of Medicine and Pharmacy, Dept. of Nephrology, Timisoara, Romania; 2 “Victor Babes” University of Medicine and Pharmacy, Dept. of Diabetes and Metabolic Diseases, Timisoara, Romania; 3 “Victor Babes” University of Medicine and Pharmacy, Department of Pharmacology, Timisoara, Romania; 4 “Victor Babes” University of Medicine and Pharmacy, Dept. of Cellular Biology, Timisoara, Romania; 5 County Emergency Hospital, Timisoara, Romania; 6 “Victor Babes” University of Medicine and Pharmacy, Dept. of Public Health Medicine, Timisoara, Romania; National Center for Scientific Research Demokritos, Greece

## Abstract

**Background:**

There is an ongoing debate as to whether early diabetic nephropathy in Type 2 diabetes mellitus may be attributed to the glomerulus or to the proximal tubule. Urinary excretion of nephrin and vascular endothelial growth factor may increase even in the normoalbuminuria stage. In the course of diabetic nephropathy, the proximal tubule may be involved in the uptake of urinary nephrin and vascular endothelial growth factor.

**Materials and Methods:**

Two groups of consecutive Type 2 diabetes mellitus outpatients (38 normo-, 32 microalbuminuric) and 21 healthy subjects were enrolled in a cross-sectional study and evaluated concerning the relation of proximal tubule dysfunction with the podocyte biomarkers excretion, assessed by ELISA methods. The impact of advanced glycation end-products on this relation was also queried.

**Results:**

Urinary alpha_1_-microglobulin and kidney injury molecule-1 correlated with urinary albumin:creatinine ratio (R^2^ = 0.269; p<0.001; R^2^ = 0.125; p<0.001), nephrinuria (R^2^ = 0.529; p<0.001; R^2^ = 0.203; p<0.001), urinary vascular endothelial growth factor (R^2^ = 0.709; p<0.001; R^2^ = 0.360; p<0.001), urinary advanced glycation end-products (R^2^ = 0.578; p<0.001; R^2^ = 0.405; p<0.001), serum cystatin C (R^2^ = 0.130; p<0.001; R^2^ = 0.128; p<0.001), and glomerular filtration rate (R^2^ = 0.167; p<0.001; R^2^ = 0.166; p<0.001); nephrinuria and urinary vascular endothelial growth factor correlated with urinary albumin:creatinine ratio (R^2^ = 0.498; p<0.001; R^2^ = 0.227; p<0.001), urinary advanced glycation end-products (R^2^ = 0.251; p<0.001; R^2^ = 0.308; p<0.001), serum cystatin C (R^2^ = 0.157; p<0.001; R^2^ = 0.226; p<0.001), and glomerular filtration rate (R^2^ = 0.087; p = 0.007; R^2^ = 0.218; p<0.001).

**Conclusions:**

In Type 2 diabetes mellitus there is an association of proximal tubule dysfunction with podocyte damage biomarkers, even in the normoalbuminuria stage. This observation suggests a potential role of the proximal tubule in urinary nephrin and urinary vascular endothelial growth factor processing in early diabetic nephropathy, a fact which could be related to advanced glycation end-products intervention. Podocyte damage and proximal tubule dysfunction biomarkers could be validated as a practical approach to the diagnosis of early diabetic nephropathy by further studies on larger cohorts.

## Introduction

Due to the increasing epidemic of diabetes mellitus (DM) worldwide, diabetic kidney disease is now the leading cause of end-stage renal disease and may be attributed up to 40% of cases referred to renal replacement therapies, both in developed and emerging countries [1], [2].

There is an ongoing debate as to whether early diabetic nephropathy (DN) in Type 2 DM may be attributed to the glomerulus or to the proximal tubule (PT). The interest for the PT involvement in the occurrence of early DN derives from the observation that patients with Type 2 DM may show a steady decline in renal function and may progress to more advanced stages of chronic kidney disease (CKD) despite the fact that they remain normoalbuminuric in the long term [3].

The classical concept concerning mechanisms of albuminuria in DN relies on defects in the glomerular filtration barrier [4], [5]. According to this hypothesis, it is considered that albuminuria is determined by the severity of the glomerular lesions, but this correlation is not strict in preceding the onset of albuminuria, a fact demonstrated on iterated renal biopsies in patients with Type 2 DM [6].

Podocytes are onthogenetic terminally-differentiated cells, located on the outer aspect of the glomerular basement membrane. Nephrin, a transmembrane protein of the immunoglobulin superfamily, is an important component of the slit diaphragm located between the foot processes of the podocytes. Its alterations lead to the limitation of the size-selectivity of the slit diaphragm [4].

The importance of nephrin as a biomarker of early DN resides in the results provided by several studies which show that increased levels of nephrinuria may be found in Type 1 and Type 2 DM patients with normoalbuminuria, a fact which demonstrates that nephrinuria may precede microalbuminuria [7], [8], [9].

Vascular endothelial growth factor (VEGF) is a pro-angiogenic factor, produced mainly by the podocytes, which acts upon these cells through an autocrine mechanism. In the early stage of DN, VEGF expression is increased within the podocytes, leading to the increase in the endothelial permeability [10] and in the autocrine effects of VEGF upon the podocytes, with subsequent proteinuria [11]. Urinary excretion of VEGF may increase even in the normoalbuminuria stage, a fact which suggests that urinary VEGF may be used as a sensitive biomarker in the diagnosis of early DN [12].

The tubular theory concerning albuminuria in the course of DM states that albuminuria is caused primarily by impaired tubular uptake of intact albumin rather than by an increased leakiness of the glomerular filtration barrier [13], [14]. In previous works performed by us in normoalbuminuric patients with Type 2 DM we demonstrated that PT dysfunction precedes the occurrence of albuminuria [15], a phenomenon which could be delayed by rosiglitazone [16] and pioglitazone [17].

Amongst other causative factors related to PT dysfunction, advanced glycation end- products (AGE) have been involved in the pathogenesis of diabetic tubulopathy, an emerging entity [18]. The aim of our study was to evaluate a potential relation of PT dysfunction with urinary nephrin and urinary VEGF excretion in normoalbuminuric patients with Type 2 DM as compared to microalbuminuric ones. Also, we queried if this association could be related to AGE intervention, which may impact both the PT and the podocytes. We found that in Type 2 DM there is an association of PT dysfunction with podocyte damage biomarkers, even in the normoalbuminuria stage. This observation suggests a potential role of the PT in urinary nephrin and urinary VEGF processing in early DN. Podocyte damage and PT dysfunction biomarkers could be validated as a practical approach to the diagnosis of early DN.

## Materials and Methods

A total of 70 consecutive patients with Type 2 DM (38 patients with normoalbuminuria – group A – and 32 patients with microalbuminuria – group B) attending the Outpatient Department of Diabetes and Metabolic Diseases and 21 healthy control subjects (group C) were enrolled in a cross-sectional study. The inclusion criteria were duration of DM higher than 5 years, normoalbuminuria [urine albumin-to-creatinine ratio (UACR)<30 mg/g] or microalbuminuria (UACR between 30 and 300 mg/g), therapy with oral antidiabetic drugs, angiotensin-converting enzyme inhibitors and/or angiotensin receptor blockers, and statins.

All patients were assessed concerning urinary alpha_1_-microglobulin and urinary kidney injury molecule-1 (KIM-1) as biomarkers of PT dysfunction; urinary nephrin and urinary VEGF, as markers of podocyte damage; plasma and urinary AGE; UACR and serum cystatin C.

Serum and urinary biomarkers were determined in specimens frozen at −80°C and thawed before assay. CKD was defined according to the KDIGO Guideline for the Evaluation and Management of CKD (2009 CKD-EPI creatinine equation) [19].

***Cystatin C*** was assessed in serum with N latex cystatin C kit (Siemens Healthcare Diagnostics, Marburg, Germany) through particle-enhanced immunonephelometry using the BNProSpec System. The reference interval was calculated nonparametrically and was determined to be 0.53–0.95 mg/l. The intra-assay precision was 2.5% coefficient of variation (CV) and inter-assay precision was 2.0% CV with a total of 2.8% CV. Analytical sensitivity was calculated as two standard deviations above the mean signal of 20 replicates of N diluent and was determined to be 0.005 mg/l. A typical detection for N latex cystatin C is 0.05 mg/l.

***Alpha_1_-microglobulin*** was evaluated in the second morning urine specimen with N α1-microglobulin kit (Siemens Healthcare Diagnostics, Marburg, Germany) through particle-enhanced immunonephelometry using the BNProSpec System. The reference interval was 12 mg/l or 0.07–5 mg/g creatinine. The intra-assay precision was 2.9–5.2% CV, while the inter-assay precision was 7.4–13.2% CV.

***KIM-1*** was assessed in the second morning urine specimen by KIM-1 ELISA test kit for the detection of KIM-1 in human urine, Cat No. H-RENA-E-001, Bio Assay Works, Ijamsville, MD, USA. A human KIM-1 antibody was utilised and the detection level was set at urinary KIM-1<0.150 ng/ml.

***Albuminuria*** was measured in the second morning urine specimen through immunonephelometry on the BNProSpec System, with N Antiserum to Human Albumin (Siemens Healthcare Diagnostics, Marburg, Germany). Microalbuminuria was defined by UACR between 30 and 300 mg/g, and normoalbuminuria by UACR<30 mg/g. The N Antiserum to Human Albumin was evaluated for the assay of urine on a BN System and yielded a Within-Run CV of 2.2% and a total CV of 2.6% with a mean of 79 mg/l. The results (ten runs, four determinations per run) were evaluated by analysis of variance. Urine cultures were negative for bacteriuria in all patients.

***Nephrin*** was assessed in the second morning urine specimen by human NPHN (Nephrin) ELISA kit, Cat No. E-EL-H1901 Elabscience Biotech Co. Ltd, Wuhan, Hubei Province, China. A human NPHN antibody was utilised. The sensitivity of the assessment showed that the minimum detectable dose of Human NPHN is 0.1 ng/ml. The detection range is 0.16–10 ng/ml. The repeatability of the test displayed a CV <10%.

***VEGF*** was assessed in the second morning urine specimen by a VEGF human ELISA kit for the detection of urinary VEGF, Cat No. ab100663, Abcam, Cambridge, MA, USA. A human VEGF antibody was utilised and the minimum detectable dose of VEGF was typically less than 10 pg/ml. The intra-assay reproducibility was <10% CV, and the inter-assay reproducibility was <12% CV.

***Plasma and urinary AGE peptides*** were assessed in two 24-hour urine samples by the ELISA method with human advanced glycosylation end-products ELISA kit (E01A0002), Shanghai Blue Gene Biotech Co., Shanghai, China. The sensitivity in this assay measured in two 24-hour urine samples was 1.0 pg/ml. This assay has high sensitivity and excellent specificity of AGE. This assay contains polyclonal antibodies which assess protein-bound AGE. The system utilized allows the assessment of both high and low molecular AGE species. No significant cross-reactivity or interference between AGE and analogues was observed.

### Statistical analysis

Clinical and biological data are presented as medians and IQR, as for variables with skewed distribution. We used ANOVA test and the Bonferroni multiple-comparison test. We used simple and multiple linear regression analysis. Stata's regress command was applied for testing this. R-square and adjusted R-square are part of the output and the last one was reported in the results presented. The p values for all hypothesis tests were two-sided, and statistical significance was set at p<0.00625. All analyses were conducted with Stata 9.2 (Statacorp, Texas, USA).

### Ethics statement

The County Emergency Hospital Timisoara Ethical Committee (Board of Human Studies) approved the protocol (approval number 3/5^th^ January 2014), and every patient provided written informed consent before enrolment.

## Results

All patients and controls were included in the analysis.

The demographic, clinical and laboratory data of the patients and control subjects are presented in Table 1. According to the study inclusion criteria, all patients were on ACEIs and/or ARBs, and statins. Therefore, these parameters were not entered in Table 1 and in uni/multivariate analyses.

**Table 1:** Clinical and biological data of the studied patients.

Parameter	Group A	Group B	Group C	p*	p**	p***	p
Number of subjects	38	32	21	–	–	–	–
Age (years)	56.5 (51–61)	58.5 (52–65)	57 (52–59)	0.417	0.899	0.479	0.635
DM duration (years)	9 (7–12)	9 (6–16)	–	0.893	–	–	0.545
BMI (kg/m^2^)	31.6 (28.5–35.2)	31.7 (27.0–35.4)	23.9 (23.6–24.9)	0.969	<0.0001	<0.0001	<0.0001
SBP (mmHg)	130 (120–140)	130 (125–135)	120 (115–120)	0.693	0.003	0.0004	0.0036
DBP (mmHg)	75 (70–80)	75 (70–80)	70 (60–75)	0.646	0.002	0.020	0.0022
Hb (g/dl)	13 (12.3–14.2)	12.8 (12.4–13.5)	14.3 (13.6–14.7)	0.603	0.002	0.0006	0.0047
Serum creatinine (mg/dl)	0.98 (0.82–1.08)	0.99 (0.92–1.13)	0.82 (0.77–0.92)	0.254	0.015	0.003	0.034
eGFR (ml/min/1.73 m^2^)	71.8 (60.9–86.9)	68.2 (58.1–83.0)	85.9 (82.6–95.3)	0.255	0.005	0.001	0.005
Glycaemia (mg/dl)	139 (117.5–196)	130.5 (105–210)	95 (85–100)	0.704	<0.0001	0.0004	0.001
HbA_1c_ (%)	7.0 (6.5–7.5)	7.2 (6.4–7.8)	5.1 (4.8–5.2)	0.889	<0.0001	<0.0001	0.0003
HbA_1c_ (mmol/mol)	53 (48–58)	55 (46–62)	32 (29–33)	0.889	<0.0001	<0.0001	0.0003
Serum cholesterol (mg/dl)	213 (182–253)	215 (191–268)	140 (135–170)	0.676	<0.0001	<0.0001	<0.0001
Triglycerides (mg/dl)	146 (108–190)	150 (121–183)	92 (85–107)	0.582	0.0004	0.0001	0.0014
hsCRP(mg/dl)	4.6 (3.3–11.5)	11.1 (9.3–16.7)	0.85 (0.8–1.2)	<0.0001	<0.0001	<0.0001	<0.0001
UACR (mg/g)	21.9 (16.2–28.1)	72.1 (42.9–103.0)	19.12 (17.82–24.45)	<0.0001	0.606	<0.0001	<0.0001
Serum cystatin C (mg/l)	0.87 (0.66–0.99)	0.92 (0.79–1.08)	0.63 (0.535–0.682)	0.061	0.0009	<0.0001	<0.0001
Urinary alpha1/creat (mg/g)	3.65 (3.39–6.18)	6.94 (4.56–9.08)	2.83 (2.75–3.21)	<0.0001	0.001	<0.0001	<0.0001
Urinary KIM-1/creat (ng/g)	71.26 (47.62–98.34)	107.58 (63.96–133.78)	43.2 (49.86–40.33)	0.004	0.003	0.0001	<0.0001
Urinary VEGF/creat (ng/g)	75.40 (41.75–110.55)	109.15 (84.70–200.80)	19.5 (7.4–35.4)	0.0009	<0.0001	<0.0001	<0.0001
Urinary nephrin/creat (mg/g)	0.11 (0.09–0.14)	0.89 (0.55–1.46)	0.036 (0.034–0.067)	<0.0001	<0.0001	<0.0001	<0.0001
Urinary AGE (pg/ml)	36.15 (32.81–56.25)	68.93 (35.21–108.20)	32.48 (30.36–34.10)	0.006	0.002	<0.0001	<0.0001
Plasma AGE (pg/ml)	373.41 (320.30–521.99)	649.88 (517.20–851.88)	280.62 (252.70–300.10)	<0.0001	0.0001	<0.0001	<0.0001

DM: diabetes mellitus; BMI: body mass index; SBP: systolic blood pressure; DBP: diastolic blood pressure; Hb: haemoglobin; eGFR: estimated glomerular filtration rate; HbA_1c_: glycated haemoglobin; hsCRP: high sensitive C reactive protein; UACR: urine albumin to creatinine ratio; alpha_1_/creat: alpha_1_-microglobulin to creatinine ratio; KIM-1/creat: kidney injury molecule-1 to creatinine ratio; VEGF/creat: vascular endothelial growth factor to creatinine ratio; nephrin/creat: nephrin to creatinine ratio; AGE: advanced glycation end-products; group A: patients with normoalbuminuria; group B: patients with microalbuminuria; group C: healthy control subjects; parameters are expressed as medians and IQR, as for variables with skewed distribution; p*: Bonferroni's t-test for the differences between groups A and B; p**: Bonferroni's t-test for the differences between groups A and C; p***: Bonferroni's t-test for the differences between groups B and C; p: ANOVA analysis of variance for the differences between groups A, B, and C; statistical significance was set at 0.00625.

Significant differences were found between groups A and B with regard to high sensitive C reactive protein (hsCRP) (p<0.0001), UACR (p<0.0001), serum cystatin C (p = 0.020), urinary alpha1-microglobulin (p<0.0001), urinary KIM-1 (p = 0.003), urinary nephrin (p<0.0001), urinary VEGF (p<0.0001), urinary AGE (p<0.0001), and plasma AGE (p<0.0001). Also, significant differences were found between groups A and C, and B and C, respectively, with regard to the same parameters, including cystatin C, as well (Table 1).

In *univariate regression analysis*, urinary alpha_1_-microglobulin and urinary KIM-1 correlated directly with UACR, nephrinuria, urinary VEGF, urinary AGE, serum cystatin C, and inversely with the estimated Glomerular filtration rate (eGFR). Nephrinuria and urinary VEGF correlated directly with urinary alpha_1_-microglobulin, urinary KIM-1, UACR, urinary AGE, serum cystatin C, and inversely with eGFR (Table 2). In *multivariate regression analysis*, urinary alpha_1_-microglobulin correlated directly with urinary KIM-1, urinary AGE, nephrinuria, urinary VEGF, serum cystatin C, and inversely with eGFR; urinary KIM-1 correlated directly with urinary alpha_1_-microglobulin, urinary AGE, nephrinuria, urinary VEGF, serum cystatin C, and inversely with eGFR; nephrinuria correlated directly with UACR, urinary AGE, and urinary VEGF, while urinary VEGF correlated directly with urinary AGE, serum cystatin C, urinary alpha_1_-microglobulin, and inversely with eGFR. Due to the fact that inflammation increases VEGF expression and podocyte injury in the course of DN, hsCRP was also introduced in multivariate analysis. After adjustment for hsCRP in multivariate analysis, the association between PT dysfunction and podocyte damage biomarkers remained significant (Table 3).

**Table 2:** Univariate regression analysis for the urinary biomarkers.

Variable	R-squared*	Coef β*	p*	R-squared^#^	Coef β^#^	p^#^	R-squared^§^	Coef β^§^	p^§^	R-squared^ƒ^	Coef β^ƒ^	p^ƒ^
**eGFR**	0.166	−0.953	<0.001	0.167	−0.053	<0.001	0.087	−0.008	0.007	0.218	−1.836	<0.001
**Cystatin C**	0.128	57.336	0.001	0.130	3.215	<0.001	0.157	0.809	<0.001	0.226	127.806	<0.001
**Urinary AGE**	0.405	0.825	<0.001	0.578	0.054	<0.001	0.251	0.008	<0.001	0.308	1.211	<0.001
**UACR**	0.125	0.427	<0.001	0.269	0.034	<0.001	0.498	0.010	<0.001	0.227	0.969	<0.001
**KIM-1/creat**	–	–	–	0.531	0.040	<0.001	0.203	0.005	<0.001	0.360	1.008	<0.001
**Urinary alpha1/creat**	0.531	13.107	<0.001	–	–	–	0.529	0.167	<0.001	0.709	25.469	<0.001
**Nephrin/creat**	0.203	35.316	<0.001	0.529	3.169	<0.001	–	–	–	0.527	95.648	<0.001
**VEGF/creat**	0.360	0.356	<0.001	0.709	0.027	<0.001	0.527	0.005	<0.001	–	–	–

KIM-1/creat: kidney injury molecule-1 to creatinine ratio; alpha1/creat: alpha_1_-microglobulin to creatinine ratio; nephrin/creat: nephrin to creatinine ratio; VEGF/creat: vascular endothelial growth factor to creatinine ratio; eGFR: estimated glomerular filtration rate; AGE: advanced glycation end-products; UACR: urine albumin-to-creatinine ratio; *: data for KIM-1/creat; ^#^: data for urinary alpha1/creat; ^§^: data for nephrin/creat; ^ƒ^: data for VEGF/creat.

**Table 3:** Multivariate regression analysis for the urinary biomarkers.

Variable	Coef β	p	95% CI	F	Prob>F	R^2^
Constant^#^	1.214	0.004	0.392 to 2.036	82.48^##^	<0.001^##^	0.887^##^
eGFR^#^	−1.666	0.006	−0.495 to −2.838			
Cystatin C^#^	1.866	<0.001	0.945 to 0.787			
Urinary AGE^#^	0.020	<0.001	0.013 to 0.028			
KIM-1/creat^#^	0.011	<0.001	0.005 to 0.018			
Nephrin/creat^#^	1.078	0.001	0.465 to 1.691			
VEGF/creat^#^	0.014	<0.001	0.009 to 0.019			
hsCRP^#^	−0.016	0.331	−0.050 to 0.017			
Constant*	1.513	0.002	0.211 to 3.098	86.31**	<0.001**	0.752**
eGFR*	−1.834	0.006	−0.204 to −3.116			
Cystatin C*	1.968	<0.001	1.098 to 3.689			
Urinary AGE*	0.063	<0.001	0.008 to 0.041			
Urinary alpha1/creat*	0.008	<0.001	0.029 to 0.018			
Nephrin/creat*	0.913	0.005	0.406 to 1.141			
VEGF/creat*	0.015	<0.001	0.012 to 0.027			
hsCRP*	−0.011	0.379	−0.039 to 0.006			
Constant^ƒ^	−38.012	0.025	−71.178 to −4.846	58.84^ƒƒ^	<0.001^ƒƒ^	0.796^ƒƒ^
eGFR^ƒ^	− 61.423	0.008	−106.517 to −16.329			
Cystatin C^ƒ^	100.334	<0.001	60.706 to 139.962			
Urinary alpha1/creat^ƒ^	28.381	<0.001	23.483 to 33.279			
Urinary AGE^ƒ^	0.461	0.011	0.813 to 0.108			
hsCRP^ƒ^	−1.020	0.074	−2.140 to 0.100			
Constant^§^	−0.420	<0.001	−0.530 to −0.311	78.56^§§^	<0.001^§§^	0.775^§§^
UACR^§^	0.006	<0.001	0.004 to 0.008			
VEGF/creat^§^	0.003	<0.001	0.002 to 0.004			
Urinary AGE^§^	0.046	<0.001	0.011 to 0.063			
hsCRP^§^	−0.011	0.468	−0.133 to 0.046			

alpha1/creat: alpha_1_-microglobulin to creatinine ratio; KIM-1/creat: kidney injury molecule-1 to creatinine ratio; VEGF/creat: vascular endothelial growth factor to creatinine ratio; nephrin/creat: nephrin to creatinine ratio; eGFR: estimated glomerular filtration rate; AGE: advanced glycation end-products; hsCRP-high sensitive C-reactive protein; UACR: urine albumin-to-creatinine ratio; ^#^: variables correlated with urinary alpha1/creat; *: variables correlated with KIM-1/creat; ^ƒ^: variables correlated with VEGF/creat; ^§^: variables correlated with nephrin/creat; ^##^: multivariate regression analysis for urinary alpha1/creat; **: multivariate regression analysis for KIM-1/creat; ^ƒƒ^: multivariate regression analysis for VEGF/creat; ^§§^: multivariate regression analysis for nephrin/creat.

## Discussion

Podocyte damage biomarkers have been discussed in relation to PT dysfunction biomarkers and emphasis has been put on potential nephrin and VEGF uptake and processing by the PT in early DN. Subsequently, AGE involvement in this setting was also queried due to the fact that AGE may impact both the podocyte and the PT.

In patients with Type 2 DM we found an association between PT dysfunction and podocyte biomarkers, a phenomenon which was independent of the level of albuminuria and of renal function.

### The biomarkers of PT dysfunction are increased in early DN and correlate with urinary AGE

The tubular theory concerning mechanisms of albuminuria in early DN states that there is a reduction in the retrieval pathway of albumin in the PT, and this impairment precedes glomerular damage [14]. AGE-modified peptides show tubulotoxic effects and lead to pathophysiologic responses described as diabetic tubulopathy [18].

Biomarkers of PT dysfunction are strongly associated to the levels of plasma and urinary AGE [20], [21]. In our study we found a significant relation of plasma and urinary AGE with biomarkers of PT dysfunction, such as urinary alpha_1_-microglobulin and urinary KIM-1.

Urinary alpha_1_-microglobulin and urinary KIM-1 are sensitive biomarkers in detecting PT dysfunction in early DN, even in normoalbuminuric patients. Data provided by several studies performed in normoalbuminuric patients with Type 2 DM, who displayed increased levels of urinary alpha_1_-microglobulin [15]–[17], [22], and of urinary KIM-1 [23] show that tubular functional defects precede the onset of albuminuria. In a study performed in patients with Type 2 DM, urinary tubular markers were independently associated with albuminuria in the early stage of DN [23].

Higher levels of urinary KIM-1 were found in Type 2 DM patients with glomerular hyperfiltration, as compared to patients with normal eGFR, showing that this glomerular phenomenon is a trigger for PT dysfunction in early DN [24].

In our study, the levels of urinary KIM-1 were increased in both normo- and microalbuminuric patients, but significantly higher in the microalbuminuric group. These data are in keeping with the results shown in the study performed by Vaidya *et al.* in patients with Type 1 DM in who lower urinary KIM-1 and N-acetyl-beta-D glucosaminidase levels were associated with the regression of microalbuminuria to normoalbuminuria. Urinary levels of the two tubular injury biomarkers were significantly elevated in patients with microalbuminuria as compared to patients with normoalbuminuria and healthy controls [25].

In our patients urinary alpha_1_-microglobulin and urinary KIM-1 correlated with UACR even at high-to-normal levels, thus raising the possibility that the PT injury may precede the onset of microalbuminuria. It is worth underlining that in our study, urinary alpha_1_-microglobulin and urinary KIM-1 correlated with urinary AGE. In a recent experimental study it has been shown that diabetic mice had a greater renal burden of AGE, which correlated with UACR, hyperfiltration, and release of urinary KIM-1 [26]. It may be assumed that AGE may impact the PT in the early stages of DN, thus explaining increased levels of urinary alpha-1 microglobulin and urinary KIM-1 in normoalbuminuric patients with Type 2 DM.

### Nephrinuria and urinary VEGF parallel PT dysfunction in patients with Type 2 DM

Nephrin is a podocyte-specific protein and its presence in the urine correlates with podocyte lesions in the course of DN. In a study conducted by Pätäri *et al.* in the urine of patients with Type 1 DM, nephrin was evidenced in 30% of normoalbuminuric patients, in 17% of those with microalbuminuria, in 28% of those with macroalbuminuria, and in 28% of those who were with recent onset albuminuria. The detection of nephrinuria in normoalbuminuric patients is highly predictive for the development of DN [7].

The results of our study substantiate the fact that nephrinuria is increased in patients with Type 2 DM, even in the normoalbuminuria stage. We found elevated levels of nephrinuria which correlated with the biomarkers of PT dysfunction, urinary AGE, UACR, cystatin C, and the eGFR. These correlations did not hold true for the healthy subjects studied, a fact which increases the predictive diagnostic power of nephrinuria in early DN.

Increased levels of nephrinuria, which paralleled the elevated levels of PT dysfunction markers, such as NGAL and heart-fatty acid binding protein, have been shown in a study conducted on an animal model in rats with streptozocin induced Type 1 DM and early DN [27].

In a study performed in patients with Type 2 DM, there has been evidenced an independent correlation between nephrinuria and UACR and eGFR, respectively. Nephrinuria was associated significantly with decreased levels of eGFR, even in normoalbuminuric patients, thus suggesting that nephrinuria may be potentially involved in the development of renal insufficiency in the stage of normoalbuminuria. Our results are in keeping with these data which point to the fact that patients with Type 2 DM and normoalbuminuria are very often perceived as a group of patients at low risk of developing CKD [8].

In another study performed in Type 2 DM patients, nephrinuria was detected in 54% of normoalbuminuric patients, suggesting its potential role as an early biomarker of DN. In this report, nephrinuria correlated significantly with albuminuria and showed a negative correlation with eGFR [9].

Although these data suggest that alterations of the glomerular filtration barrier may be present in the early stages of DN, nephrinuria is evidenced only in a reduced percentage of patients, a phenomenon which is expressed when PT dysfunction occurs. Retrieval of albumin by the PT through specific endocytotic mechanisms which utilize the megalin/cubilin receptor complex explains the delay in the occurrence of microalbuminuria even in patients with detectable nephrinuria. It has been assumed that the PT may be involved not only in albumin, but also in nephrin uptake in the course of DN, even in its early stages [8].

In our study, nephrinuria correlated with the biomarkers of PT dysfunction, a fact which forwards the hypothesis that the PT could have delayed the expression of glomerular involvement in early DN by coordinating nephrin uptake and processing.

In the study by Kim *et al.* urinary VEGF was increased in patients with Type 2 DM in the early stage of DN and correlated with albuminuria. Urinary VEGF excretion increased as DN advanced, thus suggesting that urinary VEGF is a sensitive biomarker of DN, predicting disease progression [12].

In our study, urinary VEGF correlated with nephrinuria and UACR, but also with the biomarkers of PT dysfunction, urinary AGE, cystatin C, and eGFR. As was the case with nephrinuria, these correlations were not found in the healthy controls.

We assume that this observation documents an association of PT with urinary VEGF uptake and processing, a fact substantiated by the strong correlation of the PT dysfunction biomarkers with urinary VEGF, even in normoalbuminuric patients. Most likely, as was the case with urinary nephrin, the PT interferes with the expression of podocyte damage markers in early DN.

The correlation of urinary VEGF with urinary AGE is in keeping with the observation that AGE induce increased VEGF expression by the podocytes in vivo and vitro [28]. In our patients, increased levels of urinary VEGF were present in both normo- and microalbuminuric patients, leading to the assumption that an increased AGE-induced VEGF expression by the podocytes may occur in the early stages of DN. Studies performed on kidney biopsies from diabetic mice [29] and Type 2 DM patients [30] show that increased levels of glycated albumin are associated with abnormal renal nephrin and VEGF expression, even in the early stages of DN.

### Cystatin C is associated with biomarkers of PT dysfunction and of podocyte damage in patients with Type 2 DM

In our study, UACR, urinary alpha_1_-microglobulin, urinary KIM-1, urinary nephrin, urinary VEGF, and plasma and urinary AGE correlated directly with serum cystatin C and negatively with eGFR. Other studies have also revealed that urinary PT damage biomarkers are associated with eGFR and cystatin C, independently of albuminuria [24], [31]. Our data have also documented a correlation of urinary nephrin and urinary VEGF with cystatin C and eGFR, even in normoalbuminuric patients, thus suggesting that these podocyte damage biomarkers may be associated to renal function decline independently of albuminuria, but most likely related to PT dysfunction.

It has been shown that reduced eGFR may correlate with the degree of albuminuria within normal range [15]-[17], [32]. Subtle changes in renal function in patients with Type 2 DM, as assessed by cystatin C, may parallel the degree of albuminuria, even in high-to- normal albuminuria [33]–[35], data which are similar to our findings. In our study, declined levels of eGFR were found in normoalbuminuric patients too, a fact that could have been attributed to PT dysfunction. These data are consistent with studies which provide evidence of a steady decline in renal function in normoalbuminuric patients with Type 2 DM [3], [36].

Our study has several limitations. First, the small study cohort affects the statistical power of the study. Second, this is a cross-sectional study which requires validation by a longitudinal study in order to prove causality between urinary nephrin and urinary VEGF excretion and PT dysfunction in Type 2 DM patients. Third, despite significant correlations in multivariate analysis between the biomarkers studied, residual confounding factors could have interfered interpretation of data, such as the extent and intervention of tubulointerstitial injury. The differences between groups concerning hsCRP may be attributed to multiple comorbid inflammatory conditions recorded in microalbuminuric patients with Type 2 DM.

The strength of our study relies upon the documentation of an association between biomarkers of PT dysfunction and biomarkers of glomerular damage in patients with Type 2 DM, relation which was independent of the degree of albuminuria and level of renal function.

To sum up, in patients with Type 2 DM, there is an association of PT dysfunction and podocyte damage biomarkers, even in the normoalbuminuria stage. This observation raises the possibility of a putative role of the PT in urinary nephrin and urinary VEGF excretion in early DN. Moreover, it forwards the hypothesis according to which podocyte damage and PT dysfunction may precede the onset of microalbuminuria. AGE could impact both the PT and the glomerulus in the early stages of DN, thus explaining increased levels of urinary nephrin and urinary VEGF in normoalbuminuric patients with Type 2 DM.

It is possible that in early DN, sequentially, PT dysfunction biomarkers may be elevated before the increase in podocyte damage biomarkers in the normoalbuminuria stage.

Presumably, the PT interferes with the expression of glomerular injury in early DN by coordinating albumin, as well as nephrin and VEGF uptake and processing. These data point to the fact that podocyte damage and PT dysfunction biomarkers could be validated as a practical approach to the diagnosis of early DN by further prospective studies on larger cohorts.
